# Supplementary material for: Establishment of an Improved ELONA Method for Detecting Fumonisin B1 Based on Aptamers and Hemin-CDs Conjugates
Source: Sensors (Basel). 2022 Sep 5;22(17):6714. doi: 10.3390/s22176714 (PMC9460299; doi:10.3390/s22176714)
Supplement: Supplementary file 1 [file sensors-22-06714-s001.zip › sensors-1900036-supplementary.pdf]

Establishment of an improved ELONA method for detecting fumonisin B_1 based on aptamers and hemin-CDs conjugates

Xinyue Zhao †, Jiale Gao †, Yuzhu Song, Jinyang Zhang and Qinqin Han *

Faculty of Life Science and Technology, Kunming University of Science and Technology, Kunming 650500, China
* Correspondence: hanqq@kust.edu.cn; Tel.: +86-(0871)-6593-9528
† These authors contributed equally to this work.

F10 encapsulated on enzyme labeling plate

1. Optimization of SA concentration

The aptamer to be coated on the enzyme label plate was used on the principle that the streptavidin (SA) and biotin (bio)markers could combine, so it was necessary to coat SA on the enzyme label plate first. However, since the amount of SA that can be encapsulated on the enzyme plate is limited, the concentration of SA needs to be optimized. SA was diluted with carbonate buffer to 5, 10, 15, 20, 25, and 30 μg/mL. 100 μL of different concentrations of SA was added to each microplate and 100 μL of carbonate buffer was added to another microplate as a blank control, and then incubated at 4ºC for 12h. The microplates were washed with PBST buffer 3X for 1 min each time. Add 200 μL of 5% BSA solution to the microtiter plate and incubate with 37°C, 60 rpm for 1 hour. The microplates were then washed with PBST buffer 3X. Add 100 μL of 1.2 μM bio-F10 solution to each microplate and incubate at 37°C, 60 rpm for 2.5 hours. The microplates were then washed with PBST buffer 3X for 1 min each time. A volume of 35 μL of F10-com-hemin-CDs conjugates and 65 μL of PBS were added to the bio-F10 labeled enzyme labeling plate and incubate at 37°C, 60 rpm for 1 h. The microplates were then washed with PBST buffer 3X. A volume of 100 μL of TMB substrate solution was added to each well and incubated in the dark at room temperature for 30 min. Then, 100 μL of 10% sulfuric acid solution was added to each well to terminate the reaction and the absorbance values of all groups were measured within 10 min at 450 nm. As shown in **Fig. S1A**, with the increase of SA concentration, OD450 gradually increased in tandem. When the concentration of SA reached 20 μg/mL, the value of OD450 levelled off, thus indicating that the amount of SA coated on the enzyme label plate was likely saturated at this point. Therefore, the optimal concentration of SA was determined to be 20 μg/mL.

2. Optimization of BSA concentration

To avoid nonspecific adsorption, BSA was used to completely coat the enzyme label plate. A volume of 100 μL of 20 μg/mL SA was added to the microplates and incubated for 12 hours at 4ºC. The microplates were then washed with PBST buffer 3X. BSA solutions of 0%, 1%, 2%, 3%, 4%, 5%, and 6% concentration were prepared with PBS. A volume of 200 μL of the BSA with different concentrations was added to each well and incubated on a shaking table of 60 rpm at 37°C for 1 h. The microplates were then washed with PBST buffer 3X. The subsequent steps were the same as for the optimization of the SA concentration. As shown in **Fig. S1B**, when the concentration of BSA increased gradually from 0% to 6%, the measured OD450 initially increased gradually. When the concentration of BSA was 5%, the OD450 no longer changed, which could have indicated that the plate was covered by BSA completely.

3. Optimization of the F10 concentration and incubation time

To explore the influence of F10 on the detection results, its concentration gradient was optimized. Bio-F10 was diluted to 0, 0.3, 0.6, 0.9, 1.2, and 1.5 μM, and different concentrations of bio-F10 100 μL were added to the SA coated enzyme label plates and incubated in a shaker at 37°C 60 rpm for 2.5 h. The microplates were then washed with PBST buffer 3X. The

subsequent steps were the same as for the optimization of the SA concentration. As shown in **Fig. S1C**, when the concentration of F10 was 0.3 - 1.5 μM, the OD450 value was significantly higher. It reached its highest value at 1.2 μM and then decreased. Therefore, 1.2 μM was selected as the best concentration of bio-F10.

The incubation times used were 0, 30, 60, 90, 120, 150, and 180 min, respectively. **Fig. S1D** shows that when the incubation time increased gradually, the value of OD450 increased gradually in tandem, and the color of the solution deepened. When the incubation time was 150 min, the value of OD450 no longer changed and the color of the solution no longer deepened, thus indicating that the combination had run to completion. Therefore, the incubation time was chosen to be 150 min.

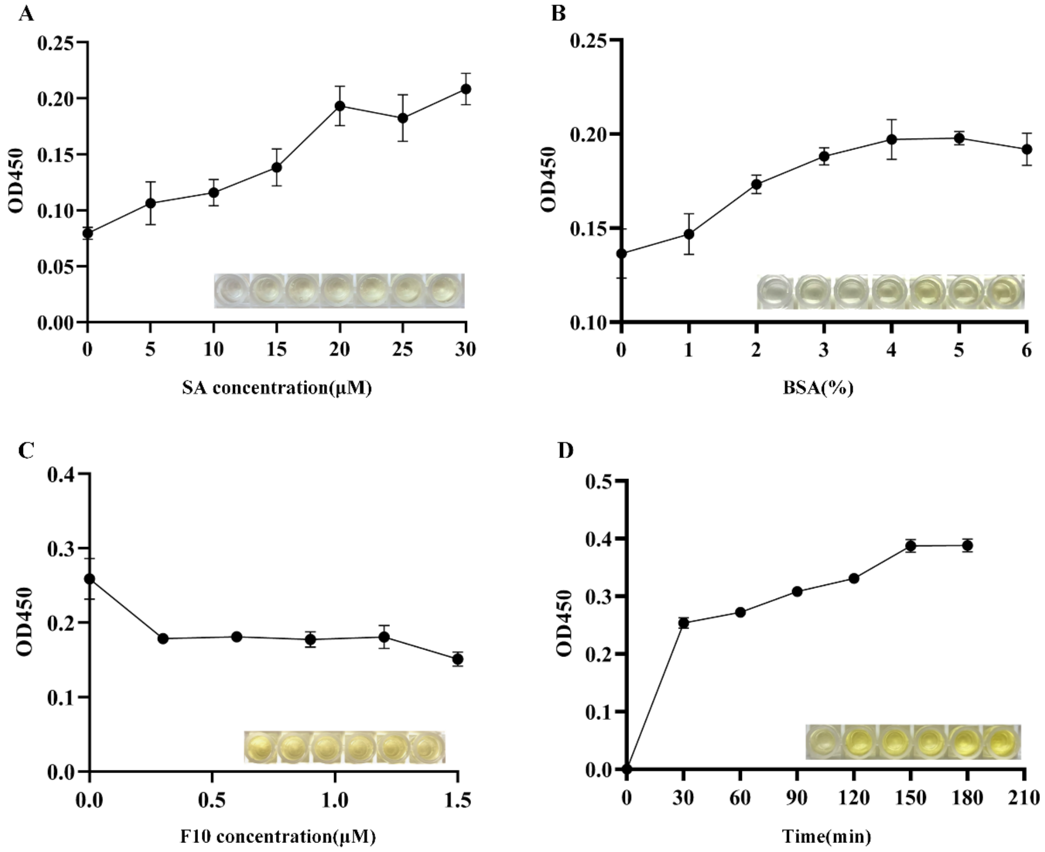

Figure S1. F10 encapsulated on enzyme labeling plate. (A) Optimization of SA concentration. (B) Optimization of BSA concentration. (C) Optimization of F10 concentration. (D) Optimization of the incubation time for F10.
